# Health care resource use by patients before and after a diagnosis of chronic fatigue syndrome (CFS/ME): a clinical practice research datalink study

**DOI:** 10.1186/s12875-017-0635-z

**Published:** 2017-05-05

**Authors:** Simon M. Collin, Inger J. Bakken, Irwin Nazareth, Esther Crawley, Peter D. White

**Affiliations:** 1 School of Social and Community Medicine, University of Bristol, Oakfield House, Oakfield Grove, Bristol, BS8 2BN UK; 2 Norwegian Institute of Public Health, PO Box 4404, Nydalen, 0403 Oslo, Norway; 3 Primary Care and Population Science, UCL Department of Primary Care and Population Health, UCL Royal Free Campus, Rowland Hill Street, London, NW3 2PF UK; 4 Psychological Medicine, Wolfson Institute of Preventive Medicine, Barts, London School of Medicine and Dentistry, Queen Mary University of London, London, UK

**Keywords:** Chronic fatigue syndrome, CFS/ME, fibromyalgia, Diagnosis, Adults, Children, Primary care, CPRD, Health care resource use

## Abstract

**Background:**

Our aim was to investigate patterns of health care resource use by patients before and after a diagnosis of CFS/ME, as recorded by Clinical Practice Research Datalink (CPRD) GP practices in the UK.

**Methods:**

We used a case–control study design in which patients who had a first recorded diagnosis of CFS/ME during the period 01/01/2001 to 31/12/2013 were matched 1:1 with controls by age, sex, and GP practice. We compared rates of GP consultations, diagnostic tests, prescriptions, referrals, and symptoms between the two groups from 15 years (in adults) or 10 years (in children) before diagnosis to 10 years after diagnosis.

**Results:**

Data were available for 6710 adult and 916 child (age <18 years) matched case–control pairs. Rates of GP consultations, diagnostic tests, prescriptions, referrals, and symptoms spiked dramatically in the year when a CFS/ME diagnosis was recorded. GP consultation rates were 50% higher in adult cases compared to controls 11–15 years before diagnosis (rate ratio (RR) 1.49 (95% CI 1.46, 1.52)) and 56% higher 6–10 years after diagnosis (RR 1.56 (1.54, 1.57)). In children, consultation rates in cases were 45% higher 6–10 years before diagnosis (RR 1.45 (1.40, 1.51)) and 62% higher 6–10 years after diagnosis (RR 1.62 (1.54, 1.70)). For adults and children, rates of tests, prescriptions, referrals, and symptoms were higher in cases compared to controls for up to 10 years before and after diagnosis.

**Conclusions:**

Adults and children with CFS/ME have greater health care needs than the rest of the population for at least ten years before their diagnosis, and these higher levels of health care resource use continue for at least ten years after diagnosis.

**Electronic supplementary material:**

The online version of this article (doi:10.1186/s12875-017-0635-z) contains supplementary material, which is available to authorized users.

## Background

Chronic fatigue syndrome (CFS, also known as ‘ME’) is a debilitating illness of unknown aetiology and pathophysiology [1]. Characterizing patients’ long-term health status before and after the onset and diagnosis of CFS/ME is important if we are to quantify the health economic and societal costs of CFS/ME, and the effects of diagnosis on health care use. Productivity losses due to CFS/ME in the UK have been estimated to be more than £100 M per annum [2]. These estimates of indirect costs were based on self-reported duration of illness (median 3 years) among patients who were able to access specialist CFS/ME services and who do not include all patients with CFS/ME. Direct costs of CFS/ME have been estimated from health care resource use by patients presenting to GPs in England, but only for short periods (3–6 months) prior to diagnosis [3, 4].

The aim of our study was to investigate long-term patterns in health care resource use before and after a diagnosis of CFS/ME using data from the Clinical Practice Research Datalink (CPRD). The CPRD provides data from approximately 7% of primary care (GP) practices in the UK [5]. We hypothesised that health care resource use would increase from around the time of patient-reported onset of illness (median self-reported duration of illness among patients who attend NHS specialist services is 3 years (adults), 16 months (children age 12–18 years) and 12 months (children <12 years old)) [1] until diagnosis. We did not have a prior hypothesis about post-diagnosis health care resource use, although this might be expected to fall if diagnosis led to referral and effective treatment. We also investigated whether health care resource use varied by sex and by socioeconomic status, given earlier evidence of a social gradient in access to CFS/ME specialist services in England [6] and in incidence of CFS/ME diagnoses [7], and we investigated adult and paediatric patients, given differences in presentation and prognosis [1].

## Methods

### Data source

The Clinical Practice Research Datalink (CPRD), formerly known as the General Practice Research Database (GPRD), is an anonymised research database aggregating medical records data from participating general practices across the UK (approximately 7% of 10,000 practices in 2012) [5]. Practices contributing to CPRD are broadly representative of general practices in the UK in terms of practice size and geographical distribution, and the source population (approximately four million ‘active’ patients, i.e. alive and registered with a GP) is broadly representative of the population of the UK in terms of age, sex, and ethnicity. GPs enter medical diagnoses and symptoms as Read codes, a hierarchical coding system used to record clinical information. Procedures, prescriptions, and referrals to secondary care are also recorded. CPRD provides two sets of data quality criteria: ‘up-to-standard’ (UTS) date for practices and ‘acceptability’ for patients [5]. These criteria do not ensure data quality, but they are used to delineate periods of quality data recording. UTS date is the date from which data in the practice are considered to be of sufficiently high quality by checking that events are being recorded continually and that recording of deaths matches projections for the practice (allowing for geographical and seasonal variation). For this study, data recorded since the practice UTS date were obtained from 660 general practices in the UK from 01/01/2001 to 31/12/2013. The acceptable patient metric is based on registration status, consistency and completeness of recording of events in the patient record, and valid information on age and sex [5]. Patients with non-contiguous records or poor data recording, which thereby raises suspicion about the validity of that patient’s record, are excluded. This study used the medical record data of patients who had acceptable data from a period beginning with the latest of the patient’s current registration period and the practice UTS date, and ending with the earliest of the patient’s transfer out date, the practice last collection date and the study end date.

### Cases

Cases were patients who had a new diagnosis of CFS/ME during the study period (01/01/2001 to 31/12/2013), as indicated by an index event with a Read code for diagnosis of CFS/ME (nine possible Read codes [8]) or referral to a CFS/ME specialist service (Additional file 1: Table S1). We considered new CFS/ME diagnoses to be those index events for which there was no preceding diagnosis of CFS/ME, FM, PVFS or asthenia/debility in the patient’s CPRD medical record as indicated by prior events with the corresponding Read codes (Additional file 1: Table S1). Read codes for referral to specialist services were introduced in 2010. Diagnoses which were made after a referral (and for which there was no prior diagnosis) were treated as new diagnoses.

### Controls

For each case, one control was matched on index event date, practice, year of birth, and sex. Controls were selected at random from patients who had no recorded diagnosis of CFS/ME, post-viral fatigue syndrome, asthenia/debility or fibromyalgia. Matching on index event date meant that the index event of the case must have occurred within the control’s registration period.

### Health care resource use

GP consultations, tests, prescriptions, and referrals were counted if they had been recorded in the patient’s medical record at any time during the patient’s current registration period with the primary care practice. Multiple GP consultations on one day were counted as a single consultation. Multiple tests per day were counted in order to reflect the total number of tests ordered. Multiple prescriptions per day were counted, but only new prescriptions were included, i.e. repeat (recurring) prescriptions were not counted. Cases and controls were required to have at least 12 months of UTS and acceptable data prior to the index event date, in order to avoid the excess events which tend to occur shortly after a patient has registered with a practice [9].

### Symptoms

Symptoms were flagged as ‘all’ symptoms, i.e. a symptom of any type, and ‘fatigue’ symptoms (if the symptom had a Read code as listed in Additional file 1: Table S1).

### Practice-level socioeconomic status

Index of Multiple Deprivation (IMD) score was used as a measure of socioeconomic status for the practice, based on its postcode. The IMD is the UK government’s official measure of deprivation.[10] It is a composite score derived from seven domains: income, employment, health and disability, education skills and training, barriers to housing and services, crime and disorder, and living environment. We grouped the top three (least deprived) quintiles and bottom two (most deprived) quintiles into two IMD strata to simplify our analysis and because our study of trends in CFS/ME diagnoses using CPRD data had shown a difference in incidence rates across these groupings [7].

### Statistical analyses

Annual rates (and 95% confidence intervals (CIs)) per patient of health care resource use events and symptoms were calculated by summing the number of events during each calendar year (for up to 15 years before and up to 10 years after the year of first recorded diagnosis of CFS/ME), and dividing by the number of patients whose medical data extended up until that time. We calculated rate ratios for cases compared with controls by fitting negative binomial regression models including a term for interaction between case status and time period, coded as three 5-year periods pre-diagnosis (11–15, 6–10 and 1–5 years before diagnosis), year of diagnosis (year 0), and two 5-year periods post-diagnosis (1–5 and 6–10 years). This allowed us to estimate the mean ratio between rates for cases compared with controls during each time period. Models were fitted using Stata (StataCorp. 2015. Stata Statistical Software: Release 14. College Station, TX: StataCorp LP).

## Results

### Study population

7901 patients had a new CFS/ME diagnosis recorded during the study period. Matched controls were available for 7626 (96.5%) of these patients. The population comprised 916 child (age <18 years) and 6710 adult patients. At the time of the index event, the median (interquartile range (IQR)) age of child patients was 15 (13–16) years, and 63.9% (585/916) were female; the median (IQR) age of adult patients was 43 (33–53) years, and 72.6% (4874/6710) were female.

The durations of pre-diagnosis data were the same in cases and controls, with a median (IQR) of 6 (3–11) years for both adult cases and controls, 8 (4–12) years for child cases, and 8 (4–11) years for child controls. Post-diagnosis data were available for 5 (3–9) years for adult cases, 5 (2–8) years for adult controls, and 4 (2–7) years for both child cases and controls. Up to 10 years of pre-diagnosis data were available for 25.9% (1737/6710) and 26.0% (1744/6710) of adult cases and controls, respectively (Additional file 1: Table S2), and 10 years of post-diagnosis data were available for 19.8% (1328/6710) and 18.4% (1232/6710) of adult cases and controls. Ten years of pre-diagnosis data were available for 32.3% (296/916) and 31.0% (284/916) of child cases and controls (Additional file 1: Table S3), and post-diagnosis data up to 10 years were available for 11.7% (107/916) and 12.1% (111/916). Fifteen years of pre-diagnosis data were available for 7.6% (509/6710) and 7.9% (529/6710) of adult cases and controls, respectively.

### Health care resource use

#### Adults

**Fig. 1:**
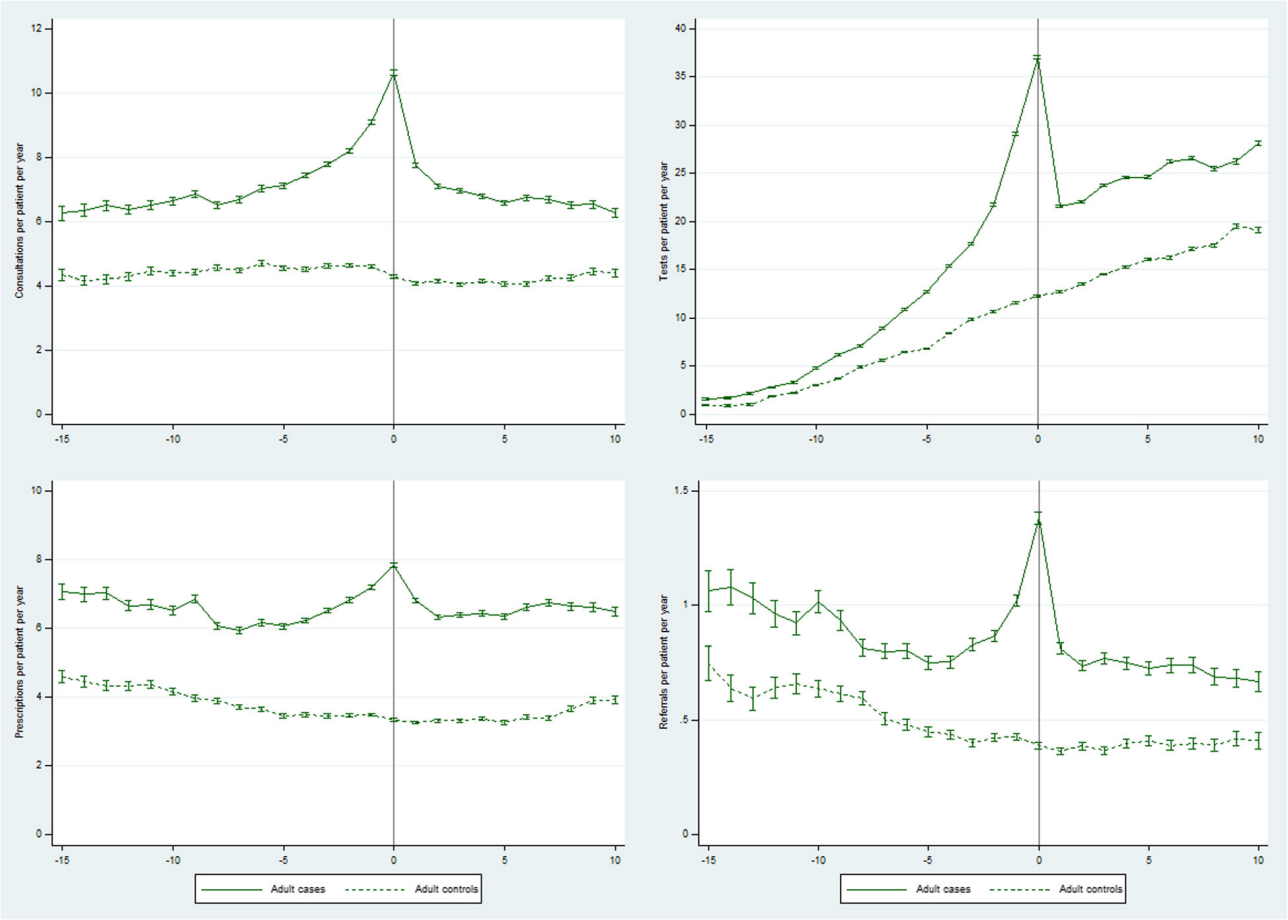
Rates of GP consultations, tests, prescriptions and referrals from 15 years before until 10 years after a first recorded diagnosis of CFS/ME in adult cases compared with controls

At 11–15 years before their first recorded CFS/ME diagnosis, GP consultations per patient per year were 49% higher in cases compared with controls (rate ratio (RR) 1.49 (95% CI 1.46, 1.52)) (Table 1, Fig. 1). During the 5 years before diagnosis, the rate of GP consultations was 76% higher (RR 1.76 (1.75, 1.77)) and, in the year when the diagnosis was recorded, the rate was 2.5 times higher (RR 2.48 (2.44, 2.51)). The rate ratio fell steeply after diagnosis, and was 56% higher (RR 1.56 (1.54, 1.57)) over the period 6–10 years post-diagnosis. Rates of tests, new prescriptions and referrals were, respectively, 64% (RR 1.64 (1.59, 1.68)), 56% (RR 1.56 (1.53, 1.58)) and 53% (RR 1.53 (1.46, 1.60)) higher in cases compared with controls at 11–15 years pre-diagnosis (Table 1, Fig. 1). Rate ratios for tests, prescriptions and referrals peaked at 3.0, 2.4 and 3.5, respectively, in the year when a CFS/ME diagnosis was first recorded. At 6–10 years post-diagnosis, rates of tests, prescriptions and referrals were, respectively, 50% (RR 1.50 (1.49, 1.51)), 85% (RR 1.85 (1.82, 1.87)) and 78% (RR 1.78 (1.72, 1.85)) higher in cases compared with controls.

**Table 1:** Rates per patient year (95% CI) and rate ratios (95% CI) of GP consultations, tests, prescriptions and referrals from 15 years before until 10 years after a first recorded diagnosis of CFS/ME in adult cases compared with controls

	Year −15 to −11	Year −10 to −6	Year −5 to −1	Year 0	Year 1 to 5	Year 6 to 10
Total person years						
Cases	4579	12,183	24,478	6710	25,447	10,815
Controls	4649	12,258	24,564	6710	25,038	10, 508
GP consultations						
Cases (rate)	6.42 (6.35, 6.49)	6.77 (6.72, 6.81)	8.06 (8.02, 8.09)	10.6 (10.6, 10.7)	7.11 (7.08, 7.14)	6.59 (6.54, 6.64)
Controls (rate)	4.31 (4.25, 4.37)	4.53 (4.49, 4.56)	4.58 (4.56, 4.61)	4.29 (4.24, 4.34)	4.10 (4.07, 4.12)	4.24 (4.20, 4.28)
Rate ratio	1.49 (1.46, 1.52)	1.50 (1.48, 1.51)	1.76 (1.75, 1.77)	2.48 (2.44, 2.51)	1.73 (1.72, 1.75)	1.56 (1.54, 1.57)
Diagnostic tests						
Cases (rate)	2.50 (2.46, 2.55)	8.01 (7.96, 8.06)	20.4 (20.3, 20.4)	37.0 (36.9, 37.2)	23.1 (23.0, 23.1)	26.4 (26.3, 26.5)
Controls (rate)	1.53 (1.49, 1.57)	4.98 (4.94, 5.02)	9.75 (9.71, 9.79)	12.3 (12.2, 12.3)	14.2 (14.1, 14.2)	17.6 (17.5, 17.7)
Rate ratio	1.64 (1.59, 1.68)	1.61 (1.59, 1.62)	2.09 (2.08, 2.10)	3.02 (3.00, 3.05)	1.63 (1.62, 1.64)	1.50 (1.49, 1.51)
Prescriptions						
Cases (rate)	6.83 (6.76, 6.91)	6.26 (6.21, 6.30)	6.63 (6.60, 6.67)	7.84 (7.78, 7.91)	6.48 (6.45, 6.51)	6.63 (6.58, 6.68)
Controls (rate)	4.39 (4.33, 4.45)	3.84 (3.81, 3.88)	3.47 (3.45, 3.50)	3.34 (3.29, 3.38)	3.31 (3.29, 3.33)	3.59 (3.56, 3.63)
Rate ratio	1.56 (1.53, 1.58)	1.63 (1.61, 1.65)	1.91 (1.89, 1.93)	2.35 (2.31, 2.39)	1.96 (1.94, 1.98)	1.85 (1.82, 1.87)
Referrals						
Cases (rate)	0.99 (0.96, 1.02)	0.86 (0.84, 0.87)	0.86 (0.85, 0.87)	1.38 (1.35, 1.41)	0.76 (0.75, 0.77)	0.71 (0.70, 0.73)
Controls (rate)	0.65 (0.63, 0.67)	0.55 (0.54, 0.57)	0.43 (0.42, 0.43)	0.39 (0.37, 0.40)	0.38 (0.37, 0.39)	0.40 (0.39, 0.41)
Rate ratio	1.53 (1.46, 1.60)	1.55 (1.50, 1.60)	2.02 (1.98, 2.07)	3.54 (3.39, 3.70)	2.00 (1.95, 2.05)	1.78 (1.72, 1.85)

#### Children

**Fig. 2:**
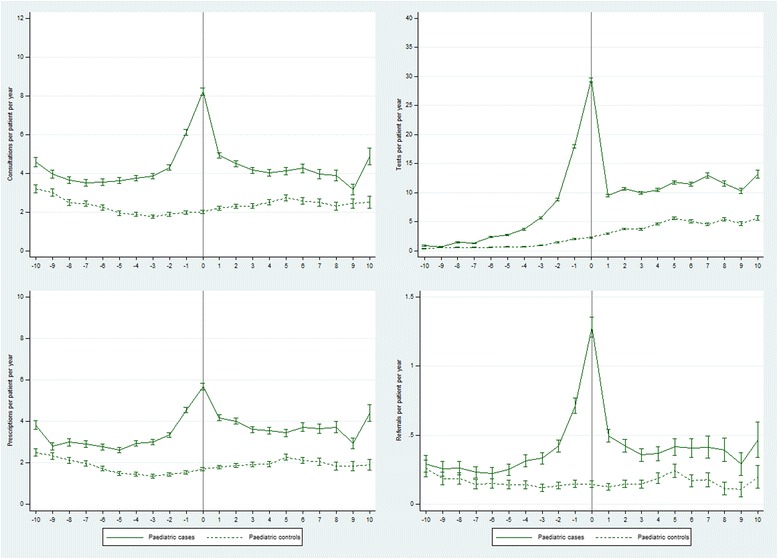
Rates of GP consultations, tests, prescriptions and referrals from 10 years before until 10 years after a first recorded diagnosis of CFS/ME in paediatric cases compared with controls

At 6–10 years before their first recorded CFS/ME diagnosis, GP consultations per patient per year were 45% higher in cases compared with controls (RR 1.45 (1.40, 1.51)) (Table 2, Fig. 2). During the 5 years before diagnosis the rate of GP consultations in cases was more than double the rate in controls (RR 2.34 (2.28, 2.41)), peaking at a 4-fold higher rate (RR 4.10 (3.90, 4.31)) in the year when the diagnosis was recorded. The rate ratio fell steeply after diagnosis, and was 62% higher (RR 1.62 (1.54, 1.70)) over the period 6–10 years post-diagnosis. Test rates in cases began almost 3-fold higher than in controls at 6–10 years pre-diagnosis (RR 2.70 (2.51, 2.89)), peaked at a 13-fold higher rate (RR 12.9 (12.3, 13.5)) and fell back to >2 times higher 6–10 years later (RR 2.37 (2.29, 2.44)) (Table 2, Fig. 2). Relative rates of new prescriptions were 44% higher 6–10 years pre-diagnosis (RR 1.44 (1.39, 1.50)), peaking at 3.3 times higher in the year of diagnosis, and falling back to 84% higher 6–10 years later (RR 1.84 (1.74, 1.93)). Referral rates were 40% higher at 6–10 years pre-diagnosis (RR 1.40 (1.22, 1.60)), peaked at a 9-fold higher rate (RR 8.83 (7.38, 10.6)) and were 2.5-fold higher 6–10 years post-diagnosis (RR 2.53 (2.12, 3.03)).

**Table 2:** Rates per patient year (95% CI) and rate ratios (95% CI) of GP consultations, tests, prescriptions and referrals from 10 years before until 10 years after a first recorded diagnosis of CFS/ME in paediatric cases compared with controls

	Year −10 to −6	Year −5 to −1	Year 0	Year 1 to 5	Year 6 to 10
Total person years					
Cases	2088	3715	916	3290	1076
Controls	1996	3591	916	3257	1077
GP consultations					
Cases (rate)	3.77 (3.69, 3.85)	4.43 (4.36, 4.49)	8.22 (8.04, 8.41)	4.42 (4.35, 4.49)	4.01 (3.89, 4.13)
Controls (rate)	2.59 (2.52, 2.66)	1.89 (1.84, 1.93)	2.01 (1.91, 2.10)	2.36 (2.31, 2.42)	2.47 (2.38, 2.57)
Rate ratio	1.45 (1.40, 1.51)	2.34 (2.28, 2.41)	4.10 (3.90, 4.31)	1.87 (1.82, 1.92)	1.62 (1.54, 1.70)
Diagnostic tests					
Cases (rate)	1.42 (1.37, 1.47)	8.44 (8.35, 8.54)	29.4 (29.1, 29.8)	10.3 (10.2, 10.4)	11.8 (11.6, 12.0)
Controls (rate)	0.53 (0.49, 0.56)	1.21 (1.17, 1.24)	2.28 (2.19, 2.38)	3.90 (3.83, 3.97)	5.00 (4.87, 5.14)
Rate ratio	2.70 (2.51, 2.89)	7.01 (6.79, 7.23)	12.9 (12.3, 13.5)	2.64 (2.59, 2.70)	2.37 (2.29, 2.44)
Prescriptions					
Cases (rate)	3.00 (2.93, 3.08)	3.37 (3.31, 3.43)	5.67 (5.52, 5.83)	3.83 (3.77, 3.90)	3.65 (3.54, 3.77)
Controls (rate)	2.08 (2.02, 2.14)	1.46 (1.42, 1.50)	1.71 (1.62, 1.79)	1.92 (1.88, 1.97)	1.99 (1.91, 2.07)
Rate ratio	1.44 (1.39, 1.50)	2.31 (2.23, 2.38)	3.33 (3.14, 3.52)	1.99 (1.93, 2.05)	1.84 (1.74, 1.93)
Referrals					
Cases (rate)	0.25 (0.23, 0.27)	0.43 (0.41, 0.45)	1.28 (1.21, 1.35)	0.42 (0.40, 0.44)	0.39 (0.36, 0.43)
Controls (rate)	0.18 (0.16, 0.20)	0.14 (0.13, 0.15)	0.15 (0.12, 0.17)	0.16 (0.15, 0.17)	0.16 (0.13, 0.18)
Rate ratio	1.40 (1.22, 1.60)	3.08 (2.79, 3.41)	8.83 (7.38, 10.6)	2.61 (2.36, 2.89)	2.53 (2.12, 3.03)

### Symptoms

#### Adults

**Fig. 3:**
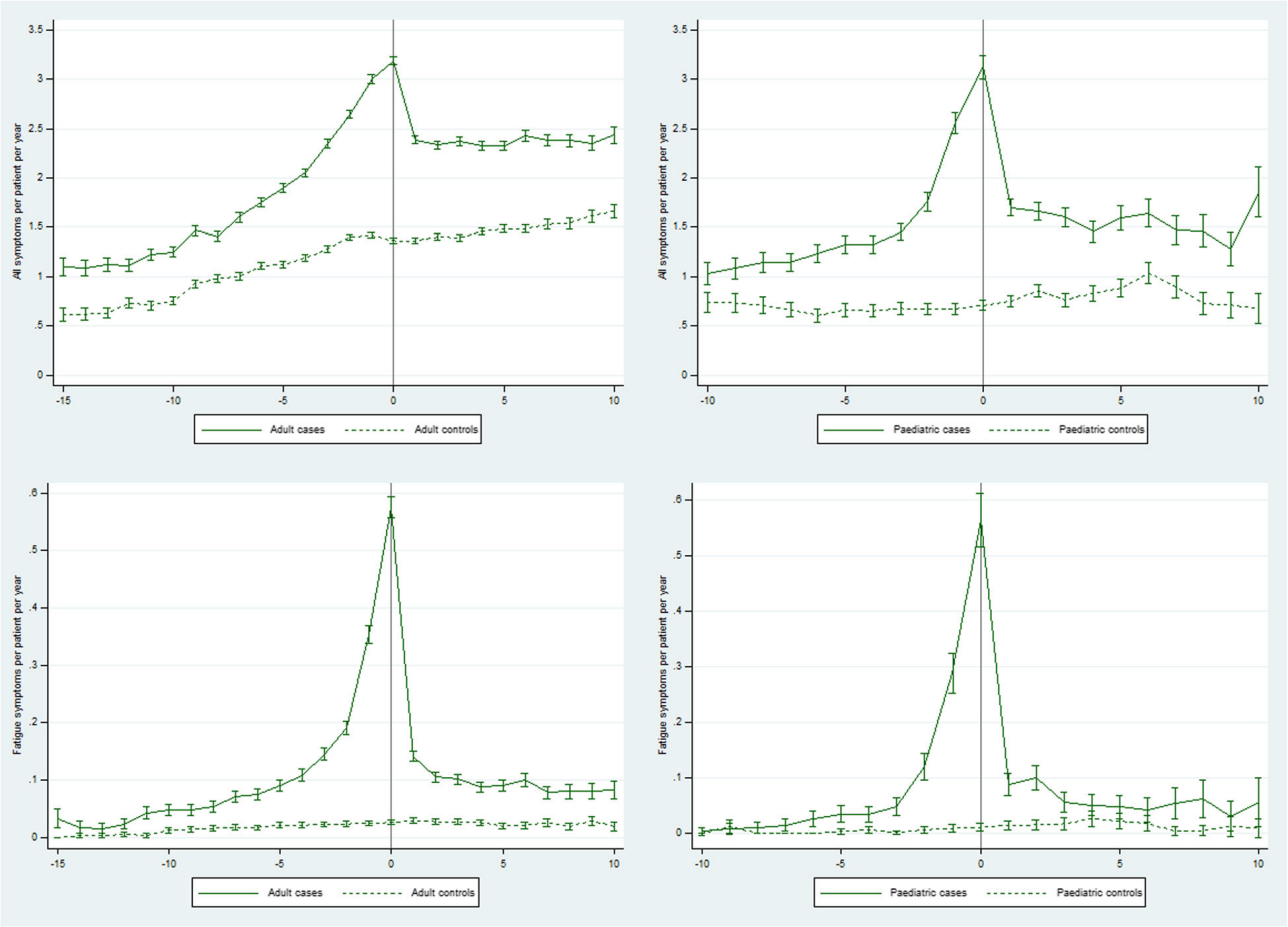
Rates of all symptoms and fatigue symptoms from 15 years before (adults) or 10 years before (paediatric) until 10 years after a first recorded diagnosis of CFS/ME in cases compared with controls

Relative rates of all (any) recorded symptoms were 55-69% higher in cases compared with controls from 6–15 years pre-diagnosis and 1–10 years post-diagnosis, peaking at a >2-fold higher rate in the year of diagnosis (RR 2.34 (2.28, 2.39)) (Table 3, Fig. 3, Additional file 1: Table S4). Relative rates of fatigue symptoms were much higher in cases compared with controls, rising from an 8-fold higher rate 11–15 years pre-diagnosis (RR 8.12 (4.83, 13.7)) to a 22-fold higher rate in the year of diagnosis (RR 21.9 (18.8, 25.5)), then falling to a 4-fold higher rate 6–10 years post-diagnosis (RR 3.75 (3.26, 4.32)).

**Table 3:** Rates (95% CI) of all symptoms and of fatigue symptoms and rate ratios (95% CI) from 15 years before (adults) or 10 years before (paediatric) until 10 years after a first recorded diagnosis of CFS/ME

	Year −15 to −11	Year −10 to −6	Year −5 to −1	Year 0	Year 1 to 5	Year 6 to 10
Total adult person years						
Cases	4579	12,183	24,478	6710	25,447	10,815
Controls	4649	12,258	24,564	6710	25,038	10, 508
Adults (all symptoms)						
Cases (rate per patient year)	1.14 (1.11, 1.17)	1.53 (1.51, 1.55)	2.46 (2.44, 2.48)	3.19 (3.15, 3.23)	2.35 (2.33, 2.37)	2.40 (2.37, 2.43)
Controls (rate per patient year)	0.67 (0.65, 0.70)	0.98 (0.96, 1.00)	1.30 (1.29, 1.32)	1.36 (1.34, 1.39)	1.41 (1.40, 1.43)	1.55 (1.53, 1.57)
Rate ratio	1.69 (1.62, 1.77)	1.57 (1.53, 1.60)	1.89 (1.87, 1.92)	2.34 (2.28, 2.39)	1.67 (1.65, 1.69)	1.55 (1.52, 1.58)
Adults (fatigue symptoms)						
Cases (rate per patient year)	2.80 (2.31, 3.28)	6.15 (5.71, 6.59)	19.4 (18.9, 20.0)	57.5 (55.7, 59.3)	10.9 (10.5, 11.3)	8.61 (8.06, 9.16)
Controls (rate per patient year)	0.34 (0.18, 0.51)	1.61 (1.38, 1.83)	2.31 (2.12, 2.50)	2.62 (2.24, 3.01)	2.66 (2.45, 2.86)	2.29 (2.00, 2.58)
Rate ratio	8.12 (4.83, 13.7)	3.83 (3.27, 4.48)	8.40 (7.70, 9.16)	21.9 (18.8, 25.5)	4.10 (3.77, 4.46)	3.75 (3.26, 4.32)
Total paediatric person years						
Cases		2088	3715	916	3290	1076
Controls		1996	3591	916	3257	1077
Paediatric (all symptoms)						
Cases (rate per 100 patient years)		1.14 (1.10, 1.19)	1.74 (1.70, 1.78)	3.12 (3.01, 3.24)	1.62 (1.58, 1.66)	1.53 (1.46, 1.61)
Controls (rate per 100 patient years)		0.68 (0.64, 0.72)	0.67 (0.64, 0.69)	0.71 (0.66, 0.76)	0.81 (0.78, 0.84)	0.86 (0.80, 0.91)
Rate ratio		1.68 (1.57, 1.79)	2.60 (2.48, 2.73)	4.40 (4.04, 4.79)	2.00 (1.91, 2.10)	1.79 (1.65, 1.94)
Paediatric (fatigue symptoms)						
Cases (rate per 100 patient years)		1.39 (0.88, 1.89)	11.6 (10.5, 12.7)	56.3 (51.5, 61.2)	7.36 (6.43, 8.28)	4.83 (3.52, 6.15)
Controls (rate per 100 patient years)		0.20 (0.00, 0.40)	0.56 (0.31, 0.80)	1.09 (0.42, 1.77)	1.81 (1.35, 2.27)	1.02 (0.42, 1.62)
Rate ratio		6.93 (2.44, 19.7)	20.8 (13.3, 32.6)	51.6 (27.6, 96.5)	4.06 (3.05, 5.40)	4.73 (2.47, 9.07)

#### Children

Symptoms of any kind were recorded at a 68% higher rate (RR 1.68 (1.57, 1.79)) in child cases compared with controls 6–10 years before diagnosis, peaking at a >4-fold higher rate in the year of diagnosis (RR 4.40 (4.04, 4.79)), and falling back to a 79% higher rate (RR 1.79 (1.65, 1.94)) 6–10 years after diagnosis (Table 3, Fig. 3, Additional file 1: Table S4). Fatigue symptoms were recorded at a 7-fold higher rate (RR 6.93 (2.44, 19.7)) in cases compared with controls 6–10 years pre-diagnosis, peaking at a 52-fold higher rate (RR 51.6 (27.6, 96.5)), and falling back to an almost 5-fold higher rate 6–10 years post-diagnosis (RR 4.73 (2.47, 9.07)).

### Variation in health care resource use by sex and socioeconomic status

**Fig. 4:**
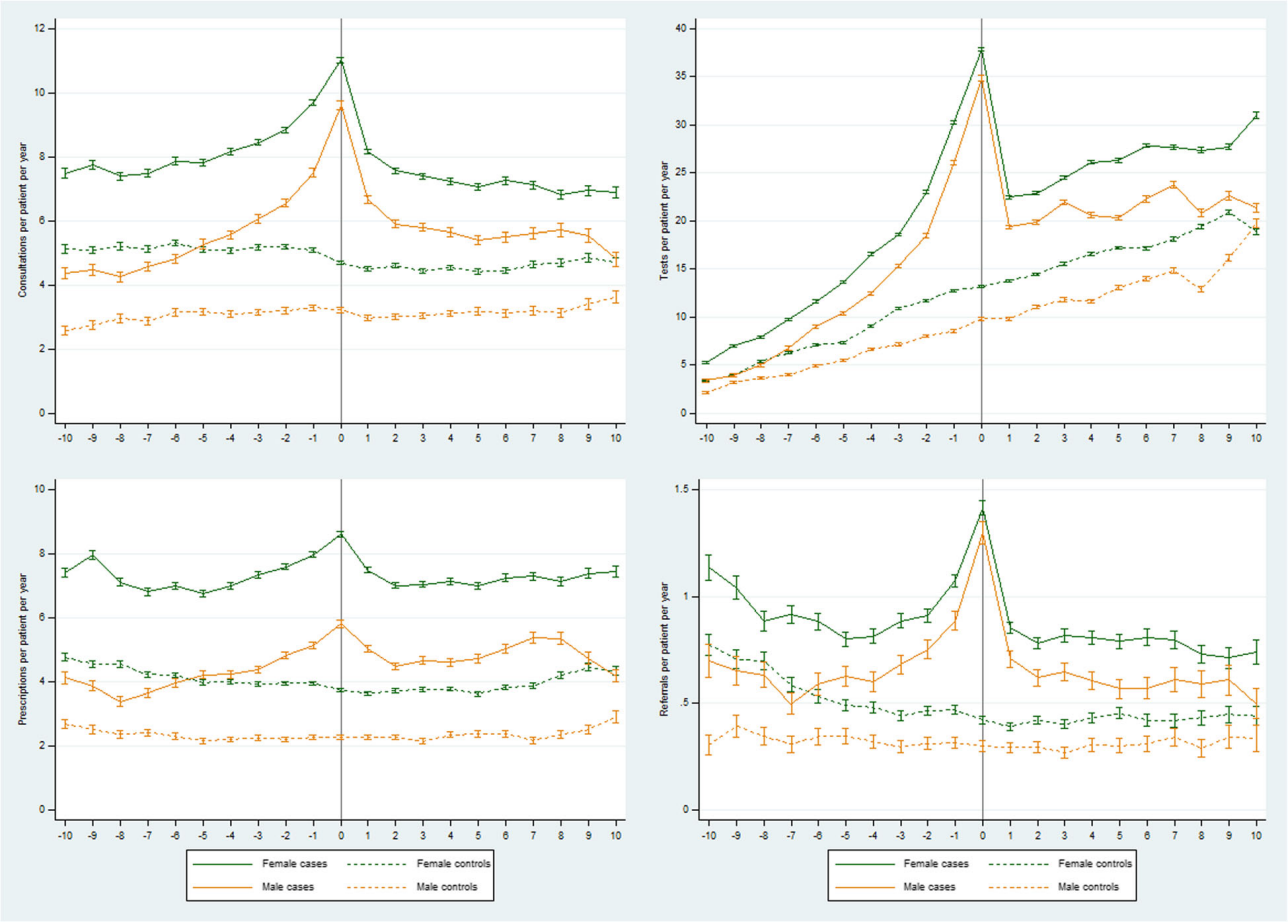
Rates of GP consultations, tests, prescriptions and referrals from 10 years before until 10 years after a first recorded diagnosis of CFS/ME in adult cases compared with controls by sex

**Fig. 5:**
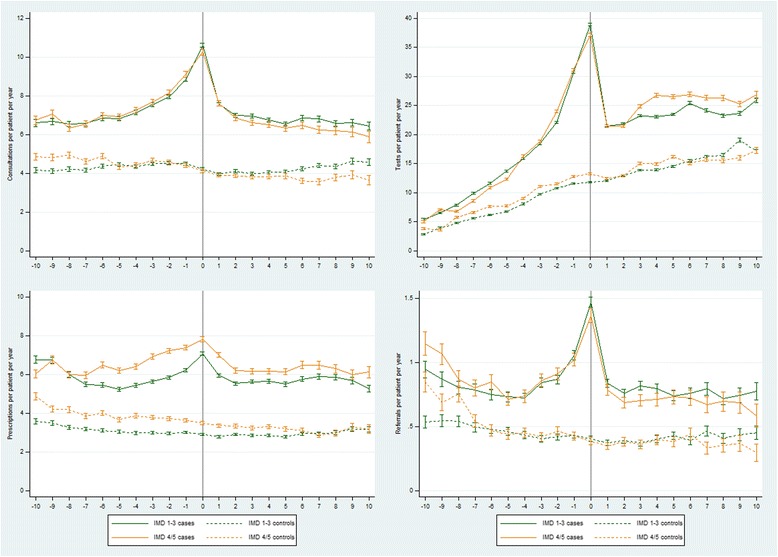
Rates of GP consultations, tests, prescriptions and referrals from 10 years before until 10 years after a first recorded diagnosis of CFS/ME in adult cases compared with controls by Index of Multiple Deprivation (IMD) quintiles

Women had higher rates of health care resource use than men (Fig. 4). Case *vs.* control rate ratios tended to be lower in female than in male patients (Table 4, Additional file 1: Tables S5a, b), but the overall patterns of relative health care resource use were similar (Fig. 4). Practice-level IMD data were available for 78.5% (5266/6710) and 85.8% (786/916) of adult and child patients, respectively. Rates of health care resource use in the upper three (least deprived) IMD quintiles were similar to rates in the lower two quintiles, although rates of prescriptions were higher in the bottom two quintiles (Fig. 5). There was little discernible difference in relative rates of health care resource use comparing cases *vs.* controls between the two socioeconomic strata (Table 5, Additional file 1: Tables S6a, b), although rate ratios for tests and prescriptions were lower in the bottom two than in the top three IMD quintiles in the pre-diagnosis periods and in the year of diagnosis.

**Table 4:** Rate ratios (95% CI) of GP consultations, tests, prescriptions and referrals from 10 years before until 10 years after a first recorded diagnosis of CFS/ME in adult cases compared with controls by sex

	Year −10 to −6	Year −5 to −1	Year 0	Year 1 to 5	Year 6 to 10
	Year −10 to −6	Year −5 to −1	Year 0	Year 1 to 5	Year 6 to 10
GP consultations					
Female	1.47 (1.45, 1.49)	1.70 (1.69, 1.71)	2.35 (2.31, 2.38)	1.68 (1.66, 1.69)	1.52 (1.50, 1.54)
Male	1.56 (1.53, 1.60)	1.99 (1.96, 2.02)	2.98 (2.90, 3.07)	1.95 (1.92, 1.99)	1.69 (1.65, 1.74)
Diagnostic tests					
Female	1.60 (1.58, 1.62)	2.01 (2.00, 2.02)	2.87 (2.85, 2.90)	1.58 (1.57, 1.59)	1.51 (1.50, 1.52)
Male	1.61 (1.58, 1.65)	2.39 (2.37, 2.42)	3.56 (3.50, 3.61)	1.80 (1.79, 1.82)	1.49 (1.47, 1.51)
Prescriptions					
Female	1.63 (1.61, 1.65)	1.87 (1.85, 1.89)	2.30 (2.26, 2.34)	1.93 (1.91, 1.95)	1.79 (1.77, 1.82)
Male	1.55 (1.51, 1.60)	2.07 (2.03, 2.11)	2.56 (2.47, 2.65)	2.07 (2.03, 2.11)	2.08 (2.03, 2.14)
Referrals					
Female	1.49 (1.44, 1.54)	1.95 (1.90, 2.00)	3.34 (3.18, 3.50)	1.95 (1.89, 2.00)	1.78 (1.71, 1.86)
Male	1.77 (1.65, 1.90)	2.30 (2.18, 2.41)	4.32 (3.93, 4.73)	2.18 (2.07, 2.30)	1.80 (1.67, 1.95)

**Table 5:** Rate ratios (95% CI) of GP consultations, tests, prescriptions and referrals from 10 years before until 10 years after a first recorded diagnosis of CFS/ME in adult cases compared with controls by Index of Multiple Deprivation (IMD) quintiles

	Year −10 to −6	Year −5 to −1	Year 0	Year 1 to 5	Year 6 to 10
GP consultations					
Top 3 quintiles	1.58 (1.55, 1.60)	1.75 (1.73, 1.77)	2.52 (2.47, 2.57)	1.75 (1.73, 1.76)	1.52 (1.50, 1.55)
Bottom 2 quintiles	1.40 (1.37, 1.43)	1.77 (1.75, 1.80)	2.49 (2.42, 2.56)	1.78 (1.76, 1.81)	1.70 (1.66, 1.75)
Diagnostic tests					
Top 3 quintiles	1.78 (1.76, 1.81)	2.20 (2.19, 2.22)	3.30 (3.27, 3.34)	1.69 (1.68, 1.70)	1.48 (1.46, 1.49)
Bottom 2 quintiles	1.39 (1.37, 1.42)	2.02 (2.00, 2.03)	2.80 (2.76, 2.84)	1.69 (1.68, 1.71)	1.68 (1.66, 1.71)
Prescriptions					
Top 3 quintiles	1.81 (1.78, 1.85)	1.92 (1.89, 1.94)	2.43 (2.38, 2.49)	2.00 (1.97, 2.02)	1.90 (1.86, 1.93)
Bottom 2 quintiles	1.50 (1.47, 1.53)	1.85 (1.82, 1.88)	2.23 (2.16, 2.30)	1.93 (1.90, 1.96)	2.07 (2.01, 2.13)
Referrals					
Top 3 quintiles	1.59 (1.52, 1.66)	2.02 (1.95, 2.09)	3.64 (3.43, 3.86)	2.03 (1.97, 2.10)	1.78 (1.69, 1.87)
Bottom 2 quintiles	1.45 (1.38, 1.53)	1.97 (1.88, 2.05)	3.53 (3.25, 3.84)	1.95 (1.86, 2.05)	1.84 (1.69, 1.99)

## Discussion

### Statement of principal findings

Our study has revealed three main features of long-term patterns in health care resource use before and after a diagnosis of CFS/ME: 1) adults and children diagnosed with CFS/ME have greater health care needs than the rest of the population for at least ten years before the diagnosis; 2) from this higher baseline, there were steep increases in use of health care resources 2–4 years before diagnosis in adults and 1–2 years before diagnosis in children; and 3) a steep drop in resource use immediately after diagnosis was followed by sustained higher levels of health care resource use for at least ten years after diagnosis. These patterns were broadly similar in women and men, and in social strata defined by IMD quintiles, and were also seen in rates of symptoms generally and fatigue symptoms specifically.

The second of these three features was not unexpected. We had hypothesised that health care resource use would increase from around the time of patient-reported onset of illness, and the dramatic peaks in health care resource use and symptoms are consistent with CFS/ME being defined clinically as a disease of new or distinct onset. However, the higher levels of health needs and symptoms predating diagnosis by up to 15 years is a somewhat unexpected finding. It conflicts with patients reporting that they were fit and well prior to the onset of their illness [11, 12] (albeit in small qualitative studies), and it raises interesting questions about the natural history of CFS/ME.

### Our study in relation to other studies

In relation to onset of illness, seven case definitions for CFS/ME have been used in clinical practice and research since the first was published in 1988, and these definitions have differed mainly in the minimum duration of fatigue and the type and number of additional symptoms [13]. That the fatigue must be of new or distinct onset was a feature of the original CDC case definition [14], and was also part of the Fukuda [15], Oxford [16], and the (original) Canadian [17] case definition. The Canadian criteria and its subsequent revision allowed for a gradual onset, but recognised that onset was typically distinct [18]. The recently-introduced US Institute of Medicine criteria for Systemic Exercise Intolerance Disease (SEID) require that the fatigue be “of new or definite onset (not lifelong)” [19], as do current UK National Institute of Health and Care Excellence (NICE) clinical guidelines [20].

The distinct peak in health care resource use that we observed matches the duration of illness reported to clinicians by patients when first assessed by specialist CFS/ME services in England. For patients assessed during the period 2004–2014, the median (IQR) duration of illness for adult patients was 3 (1–8) years, and for adolescent patients (who comprise the majority of child patients) it was 1.5 (1 to 2) years [1]. The upper and lower bounds of these IQR encompass the steep upward slope in health care resource use in each patient group, i.e. the rapid increase in health care needs is consistent with patients’ (or parents’) self-report of the time since onset.

However, the sustained higher use of health care resources and numbers of symptoms predating this onset is inconsistent with patients’ (or parents’) recollections that they (or their child) had been healthy prior to the onset of CFS/ME [11, 12], and conflicts somewhat with case definitions worded to exclude fatigue that is lifelong or not of new onset. There are several possible explanations for this discrepancy.

Firstly, people may simply have had CFS/ME for a very long time. The previously quoted durations of illness (3 years for adults, 18 months for children) were self-reported, and people with CFS/ME may be under-estimating the length of time for which they have been ill. Also, these are patients who access specialist services, who will not include all patients with CFS/ME. CFS/ME may begin as a mild form which develops quite suddenly into more severe CFS/ME, thereby setting the patient on a pathway to diagnosis. Patients’ (and parents’) recall of their health status prior to the onset of more severe symptoms could be distorted by the debilitating nature of moderate to severe CFS/ME, leading them to report having been healthy. This is particularly plausible if the milder form of CFS/ME had not prevented them from being in full-time employment or had not led to notable absences from school or college. We know that adults often persevere in employment whilst coping with the symptoms of CFS/ME for years before obtaining a diagnosis [2], and an active case finding study in three secondary schools in England led to 5% (23/461) of children whose attendance was below 80% being diagnosed with CFS/ME, giving a CFS/ME prevalence of 1% (28/2855) in the three schools in the study [21]. Also, CFS/ME is accompanied by comorbidities including depression, anxiety, chronic pain, migraine and irritable bowel disease [1], which can mask CFS/ME as the primary disorder.

Secondly, it is possible that people who develop CFS/ME have pre-existing risk factors which lead to higher rates of health care resource use. Presentation for other illnesses would be consistent with a subsequent diagnosis of CFS/ME if patients were generally more susceptible to illness, e.g. immunologically or if patients with CFS/ME have different health care seeking behaviour. A small case–control study in England reported higher GP consultation rates over three 5-year periods before a diagnosis of CFS/ME, as we observed in our study [22]. The consultations were for a wide range of symptoms, and the authors concluded that behaviour traits such as disease conviction and somatic concern could not be discounted as aetiological factors. Finally, it is possible that patients who present repeatedly and regularly to primary care are more likely to be given a diagnosis of CFS/ME by their GP.

Relative rates of health care resource use and symptoms 6–10 years after diagnosis were not substantially different from rate ratios 6–10 years before diagnosis, except perhaps for lower relative rates of diagnostic tests and (in child patients) fatigue symptoms, and higher relative rates of prescriptions and referrals post-diagnosis. The overall patterns in our study were similar to those observed in a study of fibromyalgia using data from the General Practice Research Database (GPRD, the precursor to CPRD) [23]. We are unable to determine from our data whether the type of health care resource use by patients changes following diagnosis, and we do not know how many patients in our study received specialist treatment. Receiving a diagnosis may change health care resource use, because patients have an identifiable illness which explains the fatigue and symptoms that they have been experiencing and GPs may be able to provide specific advice. Adult patients in England have access to approximately 50 NHS specialist services, many of which were established under the CFS/ME Service Investment Programme (2004–2006) [24]. These services follow NICE guidance, including specific guidelines for diagnosis, specialist care, and ongoing management, with an overall patient-centred approach to treatment [20]. Although outcomes up to 20 months after treatment are favourable for some patients [25], long-term treatment outcomes for these services have not been reported. Children have better prognosis than adults [26], but specialist service provision for is much less comprehensive, with one large paediatric service in the south west of England, and patchy or non-existent provision elsewhere in the UK.

### Strengths and limitations

The main strength of our study is that it is based on a large sample from a cohort of approximately four million patients registered with a GP, representing approximately 7% of the population in the UK [5]. Patients have a median of 9.4 years (IQR 3.4-13.9) of data [5], which gives us some (but not absolute) confidence in classifying diagnoses as ‘new’ if the patient’s medical record has no previously recorded diagnoses. Two limitations of our study are that we do not know how many cases of CFS/ME in the population are undiagnosed or misdiagnosed, or what proportion of CFS/ME cases in our study were correctly diagnosed. Although knowledge and awareness of CFS/ME in primary care has improved over the past decade [27], some GPs still lack confidence in diagnosing CFS/ME [28]. This is consistent with a relatively high rate of misdiagnosis by GPs when referring to specialist services [29, 30]. The estimated incidence of adult CFS/ME diagnoses in CPRD data in the period 2008–2010 was broadly consistent with incidence estimated from data obtained directly from NHS specialist CFS/ME services in 2009, which gives some reassurance that CFS/ME diagnoses recorded by GPs towards the end of the period covered by our study were made or confirmed by a specialist CFS/ME service [7]. However, CFS/ME diagnoses in CPRD data have yet to be validated through linkage or other means.

Two other potential limitations of our study were that people who contributed the longest durations of pre- and post-diagnosis data may not have been representative of CFS/ME patients and healthy controls, and that biases in rate ratios could be introduced at the extremes of follow-up if there were differential losses between the case–control pairs. However, there were no differential losses by age, sex and IMD quintile, and sensitivity analyses restricted to patients who had either 10 years of pre-diagnosis or 10 years of post-diagnosis data yielded rate ratios which were similar to those in our main analysis.

### Unanswered questions and future research

The extent to which the elevated health care needs of adults and children who were subsequently diagnosed with CFS/ME reflect long-term undiagnosed illness requires further investigation. Ideally this would be accomplished by means of a large long-term prospective cohort study with deep phenotyping of participants, including repeated measurements of symptoms, biological markers, psychological states, and objective measures of health and function. The sample size for such studies could be reduced by following patients who have an acute infectious illness, given that CFS/ME will be triggered in a fairly constant proportion of patients [31]. However, these patients will not represent all patients who develop CFS/ME if the illness is triggered by other events. Electronic primary care records can be used to investigate whether specific illnesses occur more frequently in patients who are subsequently diagnosed with CFS/ME [32], and to investigate what the overall higher rates of health care resource use actually represent, in terms of specific tests, prescriptions, and referrals. With regard to post-diagnosis health care resource use, cohort studies are needed to quantify long-term treatment outcomes among patients who are treated by specialist CFS/ME services after diagnosis. Qualitative methods could be used to investigate how health care needs before and after diagnosis relate to patients’ health status and quality of life.

## Conclusions

We cannot discount the possibility that patients in our study have presented over a period of 10 or more years with symptoms related to CFS/ME, even if a very long prodromal period before the onset of CFS/ME appears to be inconsistent with patients’ own reports of when their illness began and with patients describing themselves as being fit and healthy prior to onset. We are also mindful of the known heterogeneity within CFS/ME, and that patterns of health care resource use could vary substantially across different CFS/ME phenotypes. Diagnosis of CFS/ME coincided with a dramatic peak in health care resource use, and had little or no impact on health resource use in the longer term.

## Additional file

Additional file 1:
**Table S1.** READ codes defining diagnoses, referrals and fatigue symptoms. **Table S2.** GP consultations, tests, prescriptions and referrals from 15 years before until 10 years after a first recorded diagnosis of CFS/ME in adult cases and controls. **Table S3.** GP consultations, tests, prescriptions and referrals from 10 years before until 10 years after a first recorded diagnosis of CFS/ME in paediatric cases and controls. **Table S4.** Symptoms from 15 years before (adults) or 10 years before (paediatric) until 10 years after a first recorded diagnosis of CFS/ME in cases and controls. **Table S5a**. GP consultations, tests, prescriptions and referrals from 10 years before until 10 years after a first recorded diagnosis of CFS/ME in female adult cases and controls. **Table S5b.** GP consultations, tests, prescriptions and referrals from 10 years before until 10 years after a first recorded diagnosis of CFS/ME in male adult cases and controls. **Table S6a.** GP consultations, tests, prescriptions and referrals from 10 years before until 10 years after a first recorded diagnosis of CFS/ME in top 3 IMD quintile adult cases and controls. **Table S6b.** GP consultations, tests, prescriptions and referrals from 10 years before until 10 years after a first recorded diagnosis of CFS/ME in bottom 2 IMD quintile adult cases and controls (DOCX 30 kb)

## Abbreviations

CFS/ME: Chronic fatigue syndrome/myalgic encephalomyelitis
CPRD: Clinical Practice Research Datalink
FM: Fibromyalgia
GPRD: General Practice Research Database
IMD: Index of Multiple Deprivation
PVFS: Post viral fatigue syndrome
RR: Rate ratio
UTS: Up To Standard
