# Supplementary material for: Health care resource use by patients before and after a diagnosis of chronic fatigue syndrome (CFS/ME): a clinical practice research datalink study
Source: BMC Fam Pract. 2017 May 5;18:60. doi: 10.1186/s12875-017-0635-z (PMC5420108; doi:10.1186/s12875-017-0635-z)
Supplement: Additional file 1: — Table S1. READ codes defining diagnoses, referrals and fatigue symptoms. Table S2. GP consultations, tests, prescriptions and referrals from 15 years before until 10 years after a first recorded diagnosis of CFS/ME in adult cases and controls. Table S3. GP consultations, tests, prescriptions and referrals from 10 years before until 10 years after a first recorded diagnosis of CFS/ME in paediatric cases and controls. Table S4. Symptoms from 15 years before (adults) or 10 years before (paediatric) until 10 years after a first recorded diagnosis of CFS/ME in cases and controls. Table S5a. GP consultations, tests, prescriptions and referrals from 10 years before until 10 years after a first recorded diagnosis of CFS/ME in female adult cases and controls. Table S5b. GP consultations, tests, prescriptions and referrals from 10 years before until 10 years after a first recorded diagnosis of CFS/ME in male adult cases and controls. Table S6a. GP consultations, tests, prescriptions and referrals from 10 years before until 10 years after a first recorded diagnosis of CFS/ME in top 3 IMD quintile adult cases and controls. Table S6b. GP consultations, tests, prescriptions and referrals from 10 years before until 10 years after a first recorded diagnosis of CFS/ME in bottom 2 IMD quintile adult cases and controls (DOCX 30 kb) [file 12875_2017_635_MOESM1_ESM.docx]

Supplementary Table 1: READ codes defining diagnoses, referrals and fatigue symptoms

| **READ CODE** | **READ TERM** |
| --- | --- |
| CFS/ME (diagnosis) | |
| Eu46011 | [X]FATIGUE SYNDROME |
| F03y.12 | MYALGIC ENCEPHALOMYELITIS |
| F286.00 | CHRONIC FATIGUE SYNDROME |
| F286.11 | CFS - CHRONIC FATIGUE SYNDROME |
| F286.15 | MYALGIC ENCEPHALOMYELITIS |
| F286.16 | ME - MYALGIC ENCEPHALOMYELITIS |
| F286000 | Mild chronic fatigue syndrome |
| F286100 | Moderate chronic fatigue syndrome |
| F286200 | Severe chronic fatigue syndrome |
| PVFS (diagnosis) | |
| F286.12 | POSTVIRAL FATIGUE SYNDROME |
| F286.13 | PVFS - POSTVIRAL FATIGUE SYN |
| F286.14 | POST-VIRAL FATIGUE SYNDROME |
| R007400 | [D]POSTVIRAL (ASTHENIC) SYNDROME |
| 1684.13 | C/O - POSTVIRAL SYNDROME |
| R007411 | [D]POST VIRAL DEBILITY |
| Asthenia/Debility (diagnosis) | |
| Eu46000 | [X]NEURASTHENIA |
| Eu46y14 | [X]PSYCHASTHENIA |
| Eu46y15 | [X]PSYCHASTHENIA NEUROSIS |
| E205.00 | NEURASTHENIA - NERVOUS DEBILITY |
| Fibromyalgia (diagnosis) | |
| N239.00 | FIBROMYALGIA |
| N248.00 | FIBROMYALGIA |
| Referral | |
| 8HkW.00 | Referral to chronic fatigue syndrome specialist team |
| 8HlL.00 | Referral for chronic fatigue syndrome activity management |
| 8Q1..00 | Activity management for chronic fatigue syndrome |
| Fatigue (symptom) | |
| R007200 | [D]ASTHENIA NOS |
| R202.00 | [D]SENILE ASTHENIA |
| R2y3.00 | [D]DEBILITY, UNSPECIFIED |
| 168..00 | TIREDNESS SYMPTOM |
| 168..11 | FATIGUE - SYMPTOM |
| 168..12 | LETHARGY - SYMPTOM |
| 168..13 | MALAISE - SYMPTOM |
| 1682.00 | FATIGUE |
| 1683.00 | TIRED ALL THE TIME |
| 1683.11 | C/O - "TIRED ALL THE TIME" |
| 1684.00 | MALAISE/LETHARGY |
| 1684.11 | C/O - DEBILITY - MALAISE |
| 168Z.00 | TIREDNESS SYMPTOM NOS |
| E205.12 | TIRED ALL THE TIME |
| R007.00 | [D]MALAISE AND FATIGUE |
| R007000 | [D]MALAISE |
| R007100 | [D]FATIGUE |
| R007211 | [D]GENERAL WEAKNESS |
| R007300 | [D]LETHARGY |
| R007500 | [D]TIREDNESS |
| R007z00 | [D]MALAISE AND FATIGUE NOS |

Supplementary Table 2: GP consultations, tests, prescriptions and referrals from 15 years before until 10 years after a first recorded diagnosis of CFS/ME in adult cases and controls

| Years pre/post diagnosis | Number of patients (denominator) | | Number of GP consultations | | Number of diagnostic tests | | Number of prescriptions | | Number of referrals | |
| --- | --- | --- | --- | --- | --- | --- | --- | --- | --- | --- |
|  | Controls | Cases | Controls | Cases | Controls | Cases | Controls | Cases | Controls | Cases |
| -15 | 529 | 509 | 2294 | 3182 | 488 | 768 | 2430 | 3599 | 395 | 541 |
| -14 | 701 | 677 | 2917 | 4298 | 612 | 1110 | 3121 | 4731 | 448 | 731 |
| -13 | 888 | 876 | 3732 | 5688 | 868 | 1843 | 3850 | 6154 | 529 | 902 |
| -12 | 1121 | 1124 | 4806 | 7166 | 2042 | 3128 | 4840 | 7479 | 720 | 1081 |
| -11 | 1410 | 1393 | 6288 | 9067 | 3105 | 4611 | 6173 | 9315 | 927 | 1287 |
| -10 | 1744 | 1737 | 7645 | 11524 | 5214 | 8240 | 7260 | 11321 | 1112 | 1766 |
| -9 | 2051 | 2036 | 9053 | 13948 | 7529 | 12498 | 8138 | 13936 | 1261 | 1902 |
| -8 | 2422 | 2409 | 11028 | 15706 | 11819 | 17033 | 9456 | 14621 | 1443 | 1960 |
| -7 | 2805 | 2788 | 12535 | 18648 | 15636 | 24830 | 10408 | 16560 | 1416 | 2233 |
| -6 | 3236 | 3213 | 15208 | 22593 | 20871 | 34991 | 11847 | 19808 | 1548 | 2576 |
| -5 | 3713 | 3689 | 16906 | 26274 | 25227 | 47068 | 12854 | 22343 | 1670 | 2772 |
| -4 | 4261 | 4228 | 19209 | 31475 | 35703 | 65167 | 14838 | 26306 | 1859 | 3190 |
| -3 | 4846 | 4804 | 22339 | 37407 | 47711 | 84920 | 16753 | 31278 | 1946 | 3983 |
| -2 | 5492 | 5511 | 25423 | 45241 | 58538 | 119858 | 19050 | 37581 | 2324 | 4773 |
| -1 | 6252 | 6246 | 28685 | 56820 | 72414 | 181903 | 21814 | 44882 | 2668 | 6375 |
| 0 (diagnosis) | 6710 | 6710 | 28788 | 71324 | 82214 | 248480 | 22397 | 52624 | 2616 | 9268 |
| 1 | 6418 | 6509 | 26174 | 50456 | 81465 | 140607 | 20962 | 44363 | 2344 | 5293 |
| 2 | 5702 | 5826 | 23712 | 41435 | 76932 | 128513 | 18962 | 36816 | 2208 | 4298 |
| 3 | 4952 | 5041 | 20052 | 35057 | 71811 | 119864 | 16404 | 32188 | 1810 | 3881 |
| 4 | 4302 | 4363 | 17827 | 29614 | 65421 | 107246 | 14521 | 28048 | 1711 | 3269 |
| 5 | 3664 | 3708 | 14887 | 24401 | 58748 | 91212 | 11977 | 23545 | 1501 | 2691 |
| 6 | 3062 | 3082 | 12450 | 20788 | 49776 | 80823 | 10449 | 20353 | 1193 | 2278 |
| 7 | 2532 | 2577 | 10708 | 17222 | 43497 | 68493 | 8586 | 17371 | 1007 | 1910 |
| 8 | 2063 | 2125 | 8761 | 13814 | 36181 | 54220 | 7565 | 14094 | 809 | 1465 |
| 9 | 1619 | 1703 | 7189 | 11140 | 31637 | 44709 | 6314 | 11273 | 678 | 1161 |
| 10 | 1232 | 1328 | 5415 | 8325 | 23556 | 37372 | 4838 | 8602 | 507 | 888 |

Supplementary Table 3: GP consultations, tests, prescriptions and referrals from 10 years before until 10 years after a first recorded diagnosis of CFS/ME in paediatric cases and controls

| Years pre/post diagnosis | Number of patients (denominator) | | Number of GP consultations | | Number of diagnostic tests | | Number of prescriptions | | Number of referrals | |
| --- | --- | --- | --- | --- | --- | --- | --- | --- | --- | --- |
|  | Controls | Cases | Controls | Cases | Controls | Cases | Controls | Cases | Controls | Cases |
| -10 | 284 | 296 | 907 | 1356 | 91 | 258 | 711 | 1132 | 74 | 87 |
| -9 | 341 | 355 | 1023 | 1399 | 187 | 226 | 797 | 996 | 64 | 92 |
| -8 | 401 | 413 | 991 | 1504 | 222 | 591 | 855 | 1235 | 75 | 108 |
| -7 | 459 | 484 | 1112 | 1701 | 257 | 620 | 907 | 1407 | 67 | 112 |
| -6 | 511 | 540 | 1142 | 1913 | 292 | 1263 | 879 | 1496 | 77 | 122 |
| -5 | 574 | 605 | 1111 | 2188 | 373 | 1652 | 854 | 1576 | 82 | 152 |
| -4 | 634 | 672 | 1189 | 2524 | 418 | 2486 | 926 | 1985 | 90 | 212 |
| -3 | 711 | 742 | 1254 | 2846 | 638 | 4211 | 966 | 2238 | 86 | 248 |
| -2 | 793 | 817 | 1500 | 3509 | 1151 | 7211 | 1155 | 2734 | 108 | 345 |
| -1 | 879 | 879 | 1726 | 5374 | 1748 | 15807 | 1347 | 3998 | 131 | 627 |
| 0 (diagnosis) | 916 | 916 | 1837 | 7532 | 2093 | 26958 | 1562 | 5197 | 133 | 1174 |
| 1 | 889 | 891 | 1954 | 4384 | 2657 | 8474 | 1603 | 3723 | 113 | 441 |
| 2 | 789 | 798 | 1818 | 3583 | 2947 | 8522 | 1476 | 3207 | 116 | 338 |
| 3 | 641 | 653 | 1481 | 2708 | 2367 | 6486 | 1232 | 2353 | 94 | 234 |
| 4 | 524 | 529 | 1310 | 2134 | 2432 | 5533 | 1017 | 1882 | 100 | 194 |
| 5 | 414 | 419 | 1133 | 1724 | 2305 | 4924 | 941 | 1442 | 101 | 174 |
| 6 | 339 | 332 | 869 | 1415 | 1717 | 3800 | 717 | 1235 | 58 | 135 |
| 7 | 262 | 263 | 653 | 1039 | 1186 | 3408 | 539 | 960 | 47 | 109 |
| 8 | 209 | 210 | 481 | 816 | 1132 | 2434 | 387 | 783 | 24 | 83 |
| 9 | 156 | 164 | 381 | 519 | 727 | 1693 | 288 | 481 | 17 | 48 |
| 10 | 111 | 107 | 278 | 521 | 625 | 1406 | 212 | 471 | 22 | 50 |

Supplementary Table 4: Symptoms from 15 years before (adults) or 10 years before (paediatric) until 10 years after a first recorded diagnosis of CFS/ME in cases and controls

| Years pre/post diagnosis | All symptoms (adults) | | Fatigue symptoms (adults) | | All symptoms (paediatric) | | Fatigue symptoms (paediatric) | |
| --- | --- | --- | --- | --- | --- | --- | --- | --- |
|  | Controls | Cases | Controls | Cases | Controls | Cases | Controls | Cases |
| -15 | 326 | 558 | 0 | 17 |  |  |  |  |
| -14 | 434 | 734 | 2 | 12 |  |  |  |  |
| -13 | 558 | 981 | 3 | 14 |  |  |  |  |
| -12 | 820 | 1254 | 6 | 26 |  |  |  |  |
| -11 | 999 | 1699 | 5 | 59 |  |  |  |  |
| -10 | 1316 | 2171 | 22 | 83 | 210 | 305 | 0 | 1 |
| -9 | 1900 | 2987 | 30 | 97 | 250 | 385 | 4 | 3 |
| -8 | 2370 | 3390 | 39 | 129 | 284 | 473 | 0 | 4 |
| -7 | 2813 | 4465 | 50 | 197 | 305 | 554 | 0 | 7 |
| -6 | 3584 | 5643 | 56 | 243 | 310 | 666 | 0 | 14 |
| -5 | 4177 | 7004 | 80 | 334 | 380 | 803 | 2 | 21 |
| -4 | 5069 | 8676 | 92 | 460 | 413 | 889 | 4 | 23 |
| -3 | 6203 | 11278 | 110 | 699 | 482 | 1079 | 1 | 36 |
| -2 | 7687 | 14587 | 130 | 1052 | 531 | 1437 | 5 | 98 |
| -1 | 8863 | 18755 | 156 | 2210 | 592 | 2250 | 8 | 253 |
| 0 (diagnosis) | 9156 | 21395 | 176 | 3858 | 650 | 2861 | 10 | 516 |
| 1 | 8725 | 15551 | 190 | 921 | 667 | 1514 | 13 | 78 |
| 2 | 8010 | 13596 | 156 | 615 | 677 | 1327 | 12 | 80 |
| 3 | 6900 | 11970 | 136 | 511 | 491 | 1046 | 11 | 37 |
| 4 | 6280 | 10154 | 111 | 386 | 436 | 770 | 14 | 27 |
| 5 | 5436 | 8627 | 72 | 339 | 365 | 668 | 9 | 20 |
| 6 | 4553 | 7484 | 65 | 310 | 351 | 546 | 6 | 14 |
| 7 | 3875 | 6141 | 65 | 203 | 235 | 388 | 1 | 14 |
| 8 | 3180 | 5056 | 40 | 170 | 152 | 307 | 1 | 13 |
| 9 | 2618 | 4003 | 47 | 139 | 111 | 210 | 2 | 5 |
| 10 | 2049 | 3238 | 24 | 109 | 75 | 199 | 1 | 6 |

Supplementary Table 5a: GP consultations, tests, prescriptions and referrals from 10 years before until 10 years after a first recorded diagnosis of CFS/ME in female adult cases and controls

| Years pre/post diagnosis | Number of patients (denominator) | | Number of GP consultations | | Number of diagnostic tests | | Number of prescriptions | | Number of referrals | |
| --- | --- | --- | --- | --- | --- | --- | --- | --- | --- | --- |
|  | Controls | Cases | Controls | Cases | Controls | Cases | Controls | Cases | Controls | Cases |
| -10 | 1240 | 1264 | 6356 | 9458 | 4141 | 6653 | 5901 | 9369 | 958 | 1436 |
| -9 | 1457 | 1478 | 7423 | 11452 | 5653 | 10312 | 6640 | 11776 | 1027 | 1539 |
| -8 | 1716 | 1736 | 8945 | 12843 | 9271 | 13677 | 7783 | 12336 | 1198 | 1535 |
| -7 | 1993 | 2023 | 10198 | 15148 | 12427 | 19675 | 8437 | 13769 | 1165 | 1852 |
| -6 | 2308 | 2327 | 12283 | 18319 | 16302 | 27027 | 9710 | 16303 | 1228 | 2055 |
| -5 | 2647 | 2670 | 13530 | 20893 | 19428 | 36498 | 10540 | 18044 | 1301 | 2134 |
| -4 | 3051 | 3048 | 15482 | 24913 | 27686 | 50485 | 12164 | 21319 | 1471 | 2482 |
| -3 | 3478 | 3465 | 18033 | 29294 | 37977 | 64393 | 13666 | 25420 | 1539 | 3071 |
| -2 | 3959 | 3983 | 20526 | 35215 | 46264 | 91576 | 15649 | 30216 | 1846 | 3624 |
| -1 | 4519 | 4509 | 22973 | 43774 | 57614 | 136659 | 17880 | 35949 | 2122 | 4838 |
| 0 (diagnosis) | 4874 | 4874 | 22874 | 53687 | 64222 | 184515 | 18224 | 41959 | 2064 | 6886 |
| 1 | 4658 | 4721 | 20962 | 38537 | 64246 | 106008 | 16949 | 35345 | 1829 | 4027 |
| 2 | 4122 | 4225 | 18952 | 32000 | 59515 | 96754 | 15362 | 29628 | 1741 | 3305 |
| 3 | 3573 | 3642 | 15857 | 26947 | 55552 | 89234 | 13436 | 25659 | 1439 | 2978 |
| 4 | 3096 | 3148 | 14079 | 22767 | 51360 | 82222 | 11687 | 22439 | 1341 | 2534 |
| 5 | 2620 | 2651 | 11573 | 18692 | 45114 | 69753 | 9493 | 18559 | 1187 | 2092 |
| 6 | 2182 | 2182 | 9704 | 15838 | 37503 | 60770 | 8345 | 15827 | 919 | 1765 |
| 7 | 1807 | 1832 | 8393 | 13041 | 32761 | 50792 | 7004 | 13357 | 759 | 1455 |
| 8 | 1476 | 1520 | 6922 | 10357 | 28632 | 41620 | 6184 | 10855 | 639 | 1109 |
| 9 | 1159 | 1212 | 5624 | 8422 | 24211 | 33605 | 5157 | 8949 | 520 | 862 |
| 10 | 883 | 937 | 4152 | 6446 | 16670 | 29035 | 3821 | 6965 | 390 | 693 |

Supplementary Table 5b: GP consultations, tests, prescriptions and referrals from 10 years before until 10 years after a first recorded diagnosis of CFS/ME in male adult cases and controls

| Years pre/post diagnosis | Number of patients (denominator) | | Number of GP consultations | | Number of diagnostic tests | | Number of prescriptions | | Number of referrals | |
| --- | --- | --- | --- | --- | --- | --- | --- | --- | --- | --- |
|  | Controls | Cases | Controls | Cases | Controls | Cases | Controls | Cases | Controls | Cases |
| -10 | 504 | 473 | 1289 | 2066 | 1073 | 1587 | 1359 | 1952 | 154 | 330 |
| -9 | 594 | 558 | 1630 | 2496 | 1876 | 2186 | 1498 | 2160 | 234 | 363 |
| -8 | 706 | 673 | 2083 | 2863 | 2548 | 3356 | 1673 | 2285 | 245 | 425 |
| -7 | 812 | 765 | 2337 | 3500 | 3209 | 5155 | 1971 | 2791 | 251 | 381 |
| -6 | 928 | 886 | 2925 | 4274 | 4569 | 7964 | 2137 | 3505 | 320 | 521 |
| -5 | 1066 | 1019 | 3376 | 5381 | 5799 | 10570 | 2314 | 4299 | 369 | 638 |
| -4 | 1210 | 1180 | 3727 | 6562 | 8017 | 14682 | 2674 | 4987 | 388 | 708 |
| -3 | 1368 | 1339 | 4306 | 8113 | 9734 | 20527 | 3087 | 5858 | 407 | 912 |
| -2 | 1533 | 1528 | 4897 | 10026 | 12274 | 28282 | 3401 | 7365 | 478 | 1149 |
| -1 | 1733 | 1737 | 5712 | 13046 | 14800 | 45244 | 3934 | 8933 | 546 | 1537 |
| 0 (diagnosis) | 1836 | 1836 | 5914 | 17637 | 17992 | 63965 | 4173 | 10665 | 552 | 2382 |
| 1 | 1760 | 1788 | 5212 | 11919 | 17219 | 34599 | 4013 | 9018 | 515 | 1266 |
| 2 | 1580 | 1601 | 4760 | 9435 | 17417 | 31759 | 3600 | 7188 | 467 | 993 |
| 3 | 1379 | 1399 | 4195 | 8110 | 16259 | 30630 | 2968 | 6529 | 371 | 903 |
| 4 | 1206 | 1215 | 3748 | 6847 | 14061 | 25024 | 2834 | 5609 | 370 | 735 |
| 5 | 1044 | 1057 | 3314 | 5709 | 13634 | 21459 | 2484 | 4986 | 314 | 599 |
| 6 | 880 | 900 | 2746 | 4950 | 12273 | 20053 | 2104 | 4526 | 274 | 513 |
| 7 | 725 | 745 | 2315 | 4181 | 10736 | 17701 | 1582 | 4014 | 248 | 455 |
| 8 | 587 | 605 | 1839 | 3457 | 7549 | 12600 | 1381 | 3239 | 170 | 356 |
| 9 | 460 | 491 | 1565 | 2718 | 7426 | 11104 | 1157 | 2324 | 158 | 299 |
| 10 | 349 | 391 | 1263 | 1879 | 6886 | 8337 | 1017 | 1637 | 117 | 195 |

Supplementary Table 6a: GP consultations, tests, prescriptions and referrals from 10 years before until 10 years after a first recorded diagnosis of CFS/ME in top 3 IMD quintile adult cases and controls

| Years pre/post diagnosis | Number of patients (denominator) | | Number of GP consultations | | Number of diagnostic tests | | Number of prescriptions | | Number of referrals | |
| --- | --- | --- | --- | --- | --- | --- | --- | --- | --- | --- |
|  | Controls | Cases | Controls | Cases | Controls | Cases | Controls | Cases | Controls | Cases |
| -10 | 866 | 848 | 3600 | 5593 | 2406 | 4590 | 3110 | 5740 | 464 | 802 |
| -9 | 1021 | 995 | 4200 | 6633 | 4005 | 6477 | 3568 | 6708 | 557 | 864 |
| -8 | 1204 | 1172 | 5076 | 7670 | 5712 | 9214 | 3933 | 7020 | 654 | 943 |
| -7 | 1386 | 1364 | 5753 | 8982 | 7801 | 13487 | 4416 | 7479 | 687 | 1069 |
| -6 | 1626 | 1589 | 7116 | 10883 | 10011 | 18425 | 5050 | 8669 | 780 | 1192 |
| -5 | 1869 | 1850 | 8320 | 12636 | 12561 | 25415 | 5696 | 9671 | 862 | 1353 |
| -4 | 2147 | 2129 | 9326 | 15208 | 17304 | 33783 | 6407 | 11610 | 932 | 1541 |
| -3 | 2444 | 2428 | 10961 | 18325 | 23759 | 44752 | 7321 | 13702 | 991 | 2042 |
| -2 | 2793 | 2831 | 12620 | 22539 | 29988 | 62627 | 8259 | 16549 | 1175 | 2458 |
| -1 | 3198 | 3212 | 14368 | 28419 | 36862 | 98668 | 9667 | 20020 | 1385 | 3391 |
| 0 (diagnosis) | 3466 | 3466 | 14608 | 36779 | 40857 | 134955 | 10085 | 24528 | 1400 | 5093 |
| 1 | 3320 | 3361 | 13154 | 25433 | 39876 | 72317 | 9280 | 20019 | 1239 | 2811 |
| 2 | 2950 | 3010 | 12122 | 21052 | 38224 | 65676 | 8585 | 16660 | 1159 | 2294 |
| 3 | 2566 | 2594 | 10191 | 18011 | 35652 | 60317 | 7344 | 14599 | 958 | 2125 |
| 4 | 2208 | 2212 | 8920 | 14946 | 30718 | 51105 | 6291 | 12492 | 890 | 1759 |
| 5 | 1866 | 1863 | 7585 | 12193 | 27154 | 43741 | 5217 | 10244 | 801 | 1379 |
| 6 | 1548 | 1526 | 6537 | 10450 | 23930 | 38794 | 4577 | 8789 | 608 | 1160 |
| 7 | 1284 | 1270 | 5651 | 8646 | 20790 | 30664 | 3795 | 7489 | 598 | 1009 |
| 8 | 1049 | 1054 | 4564 | 6917 | 17278 | 24512 | 3146 | 6175 | 430 | 759 |
| 9 | 815 | 847 | 3766 | 5594 | 15533 | 20015 | 2606 | 4812 | 356 | 630 |
| 10 | 623 | 662 | 2849 | 4262 | 10637 | 17157 | 1967 | 3493 | 282 | 514 |

Supplementary Table 6b: GP consultations, tests, prescriptions and referrals from 10 years before until 10 years after a first recorded diagnosis of CFS/ME in bottom 2 IMD quintile adult cases and controls

| Years pre/post diagnosis | Number of patients (denominator) | | Number of GP consultations | | Number of diagnostic tests | | Number of prescriptions | | Number of referrals | |
| --- | --- | --- | --- | --- | --- | --- | --- | --- | --- | --- |
|  | Controls | Cases | Controls | Cases | Controls | Cases | Controls | Cases | Controls | Cases |
| -10 | 532 | 542 | 2581 | 3652 | 2021 | 2720 | 2591 | 3265 | 451 | 621 |
| -9 | 621 | 634 | 2982 | 4476 | 2220 | 4466 | 2617 | 4276 | 428 | 676 |
| -8 | 726 | 748 | 3588 | 4749 | 4166 | 5069 | 3040 | 4483 | 547 | 651 |
| -7 | 839 | 850 | 3869 | 5552 | 5525 | 7289 | 3230 | 5073 | 456 | 681 |
| -6 | 946 | 960 | 4612 | 6698 | 7217 | 10434 | 3800 | 6232 | 446 | 812 |
| -5 | 1081 | 1079 | 4659 | 7467 | 8308 | 13281 | 3972 | 6686 | 475 | 772 |
| -4 | 1229 | 1216 | 5430 | 8824 | 11101 | 19817 | 4760 | 7807 | 555 | 897 |
| -3 | 1367 | 1349 | 6376 | 10356 | 15166 | 25432 | 5165 | 9334 | 575 | 1159 |
| -2 | 1514 | 1509 | 6923 | 12321 | 17343 | 36231 | 5650 | 10896 | 704 | 1373 |
| -1 | 1707 | 1692 | 7506 | 15427 | 21773 | 52650 | 6212 | 12501 | 732 | 1730 |
| 0 (diagnosis) | 1800 | 1800 | 7420 | 18468 | 23913 | 66876 | 6307 | 14065 | 696 | 2458 |
| 1 | 1696 | 1732 | 6625 | 13177 | 21117 | 37178 | 5719 | 12141 | 592 | 1359 |
| 2 | 1473 | 1520 | 5700 | 10434 | 19027 | 32636 | 4936 | 9426 | 564 | 1044 |
| 3 | 1245 | 1281 | 4743 | 8473 | 18753 | 31870 | 4033 | 7891 | 451 | 903 |
| 4 | 1075 | 1098 | 4113 | 7162 | 16042 | 29392 | 3568 | 6768 | 428 | 782 |
| 5 | 902 | 920 | 3475 | 5817 | 14577 | 24374 | 2887 | 5650 | 346 | 674 |
| 6 | 736 | 759 | 2650 | 4916 | 11094 | 20437 | 2293 | 4925 | 326 | 550 |
| 7 | 582 | 615 | 2070 | 3841 | 9115 | 16185 | 1676 | 3991 | 194 | 413 |
| 8 | 469 | 484 | 1777 | 3006 | 7283 | 12728 | 1397 | 3051 | 166 | 337 |
| 9 | 348 | 370 | 1359 | 2267 | 5573 | 9348 | 1146 | 2216 | 129 | 254 |
| 10 | 245 | 265 | 893 | 1557 | 4241 | 7107 | 781 | 1624 | 73 | 155 |
